# Strategies to Identify Patient Risks of Prescription Opioid Addiction When Initiating Opioids for Pain: A Systematic Review

**DOI:** 10.1001/jamanetworkopen.2019.3365

**Published:** 2019-05-03

**Authors:** Jan Klimas, Lauren Gorfinkel, Nadia Fairbairn, Laura Amato, Keith Ahamad, Seonaid Nolan, David L. Simel, Evan Wood

**Affiliations:** 1 School of Medicine, University College Dublin, Health Sciences Centre, Belfield, Dublin, Ireland; 2 Department of Medicine, University of British Columbia, St Paul’s Hospital, Vancouver, British Columbia, Canada; 3 British Columbia Centre on Substance Use, Vancouver, British Columbia, Canada; 4 Mailman School of Public Health, Columbia University, New York, New York; 5 Department of Epidemiology, Lazio Regional Health Services, Rome, Italy; 6 Durham Veterans Affairs Medical Center, Durham, North Carolina; 7 Department of Medicine, Duke University, Durham, North Carolina

## Abstract

**Importance:**

Although prescription opioid use disorder is associated with substantial harms, strategies to identify patients with pain among whom prescription opioids can be safely prescribed have not been systematically reviewed.

**Objective:**

To review the evidence examining factors associated with opioid addiction and screening tools for identifying adult patients at high vs low risk of developing symptoms of prescription opioid addiction when initiating prescription opioids for pain.

**Data Sources:**

MEDLINE and Embase (January 1946 to November 2018) were searched for articles investigating risks of prescription opioid addiction.

**Study Selection:**

Original studies that were included compared symptoms, signs, risk factors, and screening tools among patients who developed prescription opioid addiction and those who did not.

**Data Extraction and Synthesis:**

Two investigators independently assessed quality to exclude biased or unreliable study designs and extracted data from higher quality studies. The Preferred Reporting Items for Systematic Reviews and Meta-analyses of Diagnostic Accuracy Studies (PRISMA-DTA) reporting guideline was followed.

**Main Outcomes and Measures:**

Likelihood ratios (LRs) for risk factors and screening tools were calculated.

**Results:**

Of 1287 identified studies, 6 high-quality studies were included in the qualitative synthesis and 4 were included in the quantitative synthesis. The 4 high-quality studies included in the quantitative synthesis were all retrospective studies including a total of 2 888 346 patients with 4470 cases that met the authors’ definitions of prescription opioid addiction. A history of opioid use disorder (LR range, 17-22) or other substance use disorder (LR range, 4.2-17), certain mental health diagnoses (eg, personality disorder: LR, 27; 95% CI, 18-41), and concomitant prescription of certain psychiatric medications (eg, atypical antipsychotics: LR, 17; 95% CI, 15-18) appeared useful for identifying patients at high risk of opioid addiction. Among individual findings, only the absence of a mood disorder (negative LR, 0.50; 95% CI, 0.45-0.52) was associated with a lower risk of opioid addiction. Despite their widespread use, most screening tools involving combinations of questions were based on low-quality studies or, when diagnostic performance was assessed among high-quality studies, demonstrated poor performance in helping to identify patients at high vs low risk.

**Conclusions and Relevance:**

While a history of substance use disorder, certain mental health diagnoses, and concomitant prescription of certain psychiatric medications appeared useful for identifying patients at higher risk, few quality studies were available and no symptoms, signs, or screening tools were particularly useful for identifying those at lower risk.

## Key Points

**Question:**

How can physicians identify patients with pain for whom prescription opioids can be safely prescribed?

**Findings:**

This systematic review found that a history of opioid use disorder or other substance use disorder, a mental health diagnosis, and concomitant prescription of certain psychiatric medications may be associated with an increased risk of prescription opioid addiction. However, only the absence of a mood disorder appeared useful for identifying lower risk, and assessment tools incorporating combinations of patient characteristics and risk factors were not useful.

**Meaning:**

This study suggests that there are few valid ways to identify patients who can be safely prescribed opioid analgesics.

## Teaser

This systematic review of studies examining factors associated with opioid addiction investigates whether clinically useful associations exist between opioid addiction and patient characteristics, drug characteristics, and use of common screening tools.

## Introduction

Recently, prescription opioids have been detected in up to 77% of opioid-related overdose fatalities, and the health, social, and economic costs associated with opioid use disorder (OUD) continue to increase.^1,2,3^ Importantly, the quantities of opioids prescribed in different regions have been strongly associated with higher rates of subsequent opioid overdose.^4^ Nevertheless, despite the well-recognized harms of prescription opioids, recent data suggest that the number of opioid prescriptions is remaining stable, and while the United States has seen a decrease in the national opioid prescribing rate, in 2017, prescribing rates remained very high in most US jurisdictions.^5,6^

While there is great variability in the estimates,^7,8^ a substantial proportion of persons prescribed opioids for chronic pain may subsequently develop OUD. As such, the optimal mechanism for prevention remains with the promotion of safer prescribing by health care professionals. The need for safer prescribing is also acknowledged in the recent Centers for Disease Control and Prevention pain guidelines that highlight the importance of carefully screening patients to identify those at high risk of OUD.^9^ However, patient characteristics and screening tools currently in use for predicting risk of prescription opioid addiction have not been critically assessed for diagnostic performance in a systematic review. Therefore, this review describes the incidence of prescription OUD and diagnostic accuracy of strategies used for identifying patients at high vs low risk of prescription OUD being prescribed opioids for pain.

## Methods

To identify relevant articles that examined risk factors for opioid addiction as defined by study authors, MEDLINE and Embase records from January 1946 to November 2018 were systematically searched. Search terms included *opioid-related disorders*; Medical Subject Headings terms *substance related disorders*, *pain*, and *analgesics*; and terms previously found to be useful for retrieving diagnostic studies.^10^ We also restricted eligibility to studies of opioid-naive patients newly starting opioid medications for pain and excluded studies assessing for a diagnosis of OUD among patients already receiving opioid medications. We also undertook a quality review of identified studies. Specifically, studies that evaluated prescription characteristics, patient characteristics (including a previous diagnosis of either a substance use disorder or mental health disorder), and screening tools assessing symptoms of prescription opioid addiction in the context of pain management, were considered if they met a predefined quality threshold (≥3) based on a 5-level hierarchy of evidence rating scale by Simel and Rennie.^11^ In accordance with the recommendations of the Preferred Reporting Items for Systematic Reviews and Meta-analyses for Diagnostic Test Accuracy (PRISMA-DTA) reporting guideline and Standards for Reporting Diagnostic Accuracy (STARD) reporting guideline, sources of bias for each study were also evaluated with the Quality Assessment of Diagnostic Accuracy Studies (QUADAS) tool.^12,13,14,15^ Following the policies of the University of British Columbia and Providence Healthcare Research Ethics Board, studies that use deidentified data are exempt from institutional review board approval; therefore, this study was not submitted for review.

### Data Synthesis and Analysis

The population incidence of prescription OUD after opioid prescription was estimated by collating data on opioid dependence and abuse from previous reviews on the topic (including a Cochrane Collaboration review).^7,8,16^ In brief, data on the incidence of prescription OUD in opioid-naive patients being prescribed opioids for pain was extracted from the studies that met the eligibility requirement for this review (search was not intended to assess incidence of prescription OUD [eAppendix in the Supplement]). Here, summary incidence was calculated using a random-effects estimate from the included studies and performed via Comprehensive Meta-analysis software version 3 (Biostat).^17^

For illustrative purposes, where possible, data were extracted and used to calculate likelihood ratios (LRs), sensitivity, and specificity for each individual risk factor identified for the development of prescription OUD. Full details of the search strategy, search results, study quality review, and calculation of diagnostic accuracy estimates are available in the eAppendix in the Supplement. Contingency tables (2 × 2) were constructed to estimate the LRs, sensitivity, and specificity for each risk factor or screening tool. Data were entered into Microsoft Excel spreadsheets (Microsoft Corp) predesigned to calculate the sensitivity, specificity, LRs, and their 95% confidence intervals. When a symptom, sign, or risk factor was assessed in only 1 high-quality study, the LR and 95% confidence interval were reported. When a symptom, sign, or risk factor was assessed in 2 studies, the range of LRs was reported. If a symptom, sign, or risk factor was considered in 3 or more studies, the protocol sought to pool the LR data using separate univariate random-effects meta-analysis. Likelihood ratios greater than 2.5 or less than or equal to 0.5 were considered as potentially clinically useful.

## Results

### Incidence of OUD in Pain Management

Research to date has found that the incidence of symptoms of prescription OUD varies widely in reviews of studies of patients prescribed opioids, which report that between 0.10% and 34% develop prescription OUD symptoms following an opioid prescription.^7,8,16^ This review found that the incidence of prescription OUD symptoms in high-quality (level ≥3) studies varied with the patient population, care setting, and criteria for diagnosis. For instance, prescription OUD symptoms were higher in studies of chronic pain management programs, which involved direct clinical follow-up and screening of all patients, than in database studies that relied on diagnostic codes in administrative data sets (eg, criteria from the *International Classification of Diseases, Ninth Revision* [*ICD-9*], *ICD-9–Clinical Modification* [*ICD-9-CM*], or *Diagnostic and Statistical Manual of Mental Disorders, Fourth Edition, Text Revision* [*DSM-IV-TR*]). In 2 prospective observational studies of patients in chronic pain management programs that used in-person assessments for all patients and used non-*ICD* or non-*DSM* explicit criteria (eg, aberrant behaviors, evidence of illicit substances, or abnormal findings such as presence of a nonprescribed opioid), the reported incidence of these indicators was 34% (95% CI, 26%-42%)^18^ and 28% (95% CI, 22%-36%),^19^ respectively. In contrast, the incidence of prescription OUD symptoms was lower in the 2 included large database studies that relied on physician-reported *ICD-9-CM* diagnoses to assess opioid abuse and dependence. In these studies, the reported incidence of these diagnoses was 0.10% (95% CI, 0.09%-0.11%)^20^ among commercially insured persons who received an initial opioid prescription (and who received an opioid abuse or dependence diagnosis within 2 years of filling the opioid prescription [those with OUDs], vs those who did not receive such a diagnosis within 2 years [those without OUDs]), and 3.2% (95% CI, 3.0%-3.3%) among recipients of chronic opioid therapy,^21^ respectively. When subjected to a meta-analysis, across 3 studies (1 study’s data were missing) of patients prescribed opioids for pain, the incidence of prescription OUD was 2.5% (95% CI, 0.15%-30%; *I*^2^ = 99%).^18,20,21^ The heterogeneity was created by 2 very large analyses of insurance databases with low incidence (0.10%^20^ and 3.2%^21^) that dominated the results of a small prospective cohort study of patients with chronic pain referred to a psychology practice (incidence, 34%).^18^ While further research is required, these findings suggest that incidence studies relying on conventional physician reporting that is based on overall judgment (ie, a coded diagnosis from an encounter form) and incomplete surveillance may substantially underestimate the incidence of prescription OUD.

### Risk Factors for Prescription Opioid Addiction

Of 1287 identified citations that were screened, 102 were identified as being eligible for full text review, 96 of which were excluded for either not meeting the predefined study quality threshold or other reasons (eFigure in the Supplement). Overall, 6 high-quality articles were included in the qualitative (narrative) synthesis and 4 were included in the quantitative synthesis. Two studies reported on risk factors (eTable 1 in the Supplement) and 2 studies reported on risk prediction tools (eTable 2 in the Supplement). The 4 high-quality studies included in the quantitative synthesis were all retrospective studies including a total of 2 888 346 patients with 4470 cases that met the authors’ definitions of prescription opioid addiction.^18,19,20,21^ Three of the 4 high-quality studies included in the quantitative synthesis examined chronic pain,^18,19,21^ while 1 study^20^ included patients “who had at least one opioid prescription claim,” of whom 58.83% also had a diagnosis of chronic pain disorder per ICD-9 criteria (Bryan Cochran, PhD, email communication, March 7, 2019).

Among included studies that met the quality threshold (see quality assessment in eTable 3 and eTable 4 in the Supplement), a history of any pain disorder (LR, 23; 95% CI, 18-29) was associated with increased risk of prescription opioid addiction (Table 1).^20,21^ Furthermore, personality disorder (LR, 27; 95% CI, 18-41),^20^ somatoform disorder (LR, 12; 95% CI, 7.18-18),^20^ psychotic disorder (LR, 11; 95% CI, 8.5-14),^20^ and nonopioid substance use disorder (LR range, 4.2-17)^20,21^ were associated with the greatest likelihood of subsequent symptoms of prescription opioid addiction (eTable 5 and eTable 6 in the Supplement). Using a conservative estimate of pretest probability of 2.5% prescription OUD, patients with a personality disorder, somatoform disorder, or psychotic disorder would have an elevated posttest probability of 41%, 24%, and 22%, respectively, of developing OUD. While modest LRs were found for any mood disorder (LR, 6.0; 95% CI, 5.8-6.2) and any anxiety disorder (LR, 5.3; 95% CI, 5.0-5.6), only the absence of a mood disorder (negative LR, 0.50; 95% CI, 0.45-0.52) appeared to meaningfully reduce the likelihood of prescription opioid addiction.^20^ In 1 study, having a previous history of OUD was associated with an increased risk for the subsequent development of prescription OUD following opioid prescribing for chronic pain management (LR range, 17-22).^21^ Among 2 included studies, men (LR range, 1.1-1.4) were not obviously more likely than women to develop prescription OUD.^20,21^

**Table 1.** Factors Associated With Prescription Opioid Use Disorder Among Opioid-Naive Patients Initiating Prescription Opioids

Factor^a^	Source	Studies, No.	Sensitivity (95% CI)	Specificity (95% CI)	LR Positive (95% CI)	LR Negative (95% CI)
Patient mental health history						
Any personality disorder	Cochran et al,^20^ 2014	1	0.08 (0.05-0.12)	1.0 (1.0-1.0)	27 (18-41)	0.99 (0.99-1.0)
Any pain disorder	Cochran et al,^20^ 2014	1	0.02 (0.02-0.03)	1.0 (1.0-1.0)	23 (18-29)	0.98 (0.98-0.99)
Past opioid use disorder^b^	Edlund et al,^21^ 2010	1	0.07-0.09^c^	1.0-1.0^c^	17-22^c^	0.91-0.93^c^
Somatoform disorders	Cochran et al,^20^ 2014	1	0.08 (0.05-0.11)	1.0 (1.0-1.0)	12 (7.8-18)	0.99 (0.99-1.0)
Psychotic disorders	Cochran et al,^20^ 2014	1	0.19 (0.15-0.25)	1.0 (1.0-1.0)	11 (8.5-14)	0.98 (0.98-0.99)
Any mood disorder	Cochran et al,^20^ 2014	1	0.55 (0.53-0.56)	0.91 (0.91-0.91)	6.0 (5.8-6.2)	0.50 (0.45-0.52)
Any anxiety disorder	Cochran et al,^20^ 2014	1	0.29 (0.27-0.31)	0.95 (0.95-0.95)	5.3 (5-5.6)	0.75 (0.74-0.77)
Past nonopioid substance use disorder^b^	Cochran et al,^20^ 2014; Edlund et al,^21^ 2010	2	0.14-0.58^c^	0.95-0.98^c^	4.2-17^c^	0.44-0.88^c^
Patient demographic characteristics						
Male^b^	Cochran et al,^20^ 2014; Edlund et al,^21^ 2010	2	0.33-0.60^c^	0.56-0.72^c^	1.1-1.4^c^	0.72-0.96^c^
Prescription characteristics						
Concomitant medication	Cochran et al,^20^ 2014	1				
Atypical antipsychotic	Cochran et al,^20^ 2014	1	0.24 (0.22-0.25)	0.10 (0.10-0.10)	17 (15-18)	0.77 (0.76-0.79)
Anxiolytics^d^	Cochran et al,^20^ 2014	1	0.08 (0.07-0.09)	0.99 (0.99-0.99)	7.3 (6.5-8.3)	0.93 (0.92-0.94)
Tricyclics	Cochran et al,^20^ 2014	1	0.40 (0.38-0.06)	0.92 (0.92-0.92)	5.1 (4.8-5.3)	0.66 (0.64-0.68)
Anticonvulsant	Cochran et al,^20^ 2014	1	0.34 (0.32-0.35)	0.93 (0.93-0.93)	5.0 (4.8-5.3)	0.71 (0.69-0.73)
Other antidepressants	Cochran et al,^20^ 2014	1	0.45 (0.44-0.47)	0.88 (0.88-0.88)	3.8 (3.7-4.0)	0.62 (0.60-0.64)
Benzodiazepine	Cochran et al,^20^ 2014	1	0.53 (0.51-0.54)	0.81 (0.81-0.81)	2.7 (2.6-2.8)	0.59 (0.58-0.61)
Any opioid, ie, all schedule types^b^^,^^e^	Edlund et al,^21^ 2010	1	0.05-0.06^c^	0.99-0.99^c^	3.5-4.9^c^	0.95-0.96^c^
Opioid dose >120 mg/d^b^	Edlund et al,^21^ 2010	1	0.20-0.21^c^	0.94-0.94^c^	3.2-3.4^c^	0.85-0.85^c^

Abbreviation: LR, likelihood ratio.

^a^ An LR 2.5 or greater or an LR less than 0.5 was considered potentially clinically useful, and only select factors are reported in this table. See eTable 7 in the Supplement for the full list of eligible factors and calculated LRs.

^b^ The LR range is derived from 2 separate databases described in this study.^21^

^c^ Values are expressed as a range.

^d^ This category includes buspirone hydrochloride (Bryan Cochran, PhD, email communication, March 8, 2019).

^e^ Patients received at least 30 days supply of any opioid, ie, schedule III or IV AND short-acting schedule II AND long-acting schedule II opioids within a 6-month period.

Certain opioid prescription characteristics may also be associated with an increased risk of developing OUD. Specifically, a new prescription for any opioid for 30 days or more appeared to place patients at greater risk for prescription OUD when compared with those patients receiving a supply of opioids for less than 30 days (LR range, 3.5-4.9).^21^ This finding was consistent whether the opioids were prescribed alone, in tandem with schedule II short-acting opioids, or in tandem with opioids of another schedule. When patients’ opioid doses increased to greater than 120 morphine milligram equivalents per day (LR range, 3.2-3.4), they had a higher risk of subsequent prescription OUD.^21^ Although the presence of any concomitant psychiatric medication, such as anxiolytics (LR, 7.3; 95% CI, 6.5-8.3), appeared to be associated with greater risk of developing prescription OUD, as shown in Table 1, atypical antipsychotics appeared to be associated with the highest risk (LR, 17; 95% CI, 15-18).

Patients who received an opioid for less than 30 days (negative LR range, 0.95-0.96) or received a dose that did not increase to more than 120 morphine milligram equivalents per day (negative LR range, 0.85-0.85; the LR range is derived from 2 separate databases described in this study) did not have lower risk of prescription OUD.^20^ Similarly, patients receiving no concomitant psychiatric medications (negative LR ranging from 0.59 for benzodiazepines to 0.93 for anxiolytics) did not have lower risk of prescription OUD (Table 1 and eTable 7 in the Supplement).^20^

### Risk Assessment Tools

While this review identified 31 studies that have evaluated risk assessment tools for prescription OUD, only 2 studies (which examined 5 individual tools) met criteria to be defined as high quality and, thus, were included (Table 2).^18,19^ Furthermore, despite risk assessment tools becoming a widespread aspect of the prescribing of opioids in some pain management settings,^22^ their ability to identify patients at high vs low risk was limited. Among the tools evaluated through high-quality studies, the Pain Medication Questionnaire (PMQ) (cutoff score ≥30) appeared most promising, although its ability to differentiate patients at high risk from those at low risk was poor overall (Table 2). Among the PMQ validation sample, having 30 or more positive answers modestly increased the likelihood of symptoms of prescription OUD (LR, 2.6; 95% CI, 1.4-4.8). However, having fewer than 30 positive answers had marginal utility for identifying those least likely to develop prescription OUD (negative LR, 0.75; 95% CI, 0.60-0.94). In addition to its limited ability to discern patients at high risk from those at low risk and the fact that the PMQ is only useful for patients who have had pain treated in the past, the tool may be cumbersome, requiring approximately 10 minutes to administer. Similarly, no scale proved useful for assessing patients newly presenting with chronic or acute pain. Nevertheless, the PMQ is different from other scales because it assesses patient agreement with statements about their beliefs concerning past pain relief and behaviors in addressing pain, rather than traditional risk factors such as prior substance use disorders or comorbid conditions. Examples of questions include items such as “In the past, I have had some difficulty getting the medication I need from my doctor(s)” and “At times, I take pain medication when I feel anxious and sad, or when I need help sleeping.”

**Table 2.** Risk of Prescription Opioid Use Disorder Among Opioid-Naive Patients Initiating Prescription Opioids

Measure or Instrument^a^	Source	Studies, No.	Sensitivity (95% CI)	Specificity (95% CI)		LR Positive (95% CI)		LR Negative (95% CI)	
Pain Medication Questionnaire score ≥30	Jones et al,^18^ 2015	1	0.35 (0.23-0.51)		0.86 (0.78-0.92)		2.6 (1.4-4.8)		0.75 (0.60-0.94)
Opioid Risk Tool score ≥8	Jones et al,^18^ 2015	1	0.25 (0.14-0.40)		0.83 (0.74-0.90)		1.5 (0.76-2.9)		0.90 (0.75-1.1)
Brief Risk Questionnaire score ≥3	Jones et al,^18^ 2015	1	0.73 (0.52-0.85)		0.40 (0.30-0.51)		1.2 (0.96-1.6)		0.67 (0.40-1.1)
Brief Risk Interview score ≥1^b^	Jones et al,^18^ 2015	1	0.69 (0.54-0.81)		0.45 (0.34-0.55)		1.2 (0.96-1.6)		0.70 (0.43-1.1)
Screener and Opioid Assessment for Patients With Pain score ≥8^c^	Akbik et al,^19^ 2006	1	0.59 (0.49-0.68)		0.48 (0.42-0.55)		1.2 (0.94-1.4)		0.85 (0.65-1.1)

Abbreviation: LR, likelihood ratio.

^a^ An LR of 2.5 greater or less than 0.5 was considered potentially clinically useful, and only select tools from higher-quality studies are reported in this table. See eTable 2 in the Supplement for the full list of screening tools.

^b^ Positive test indicated by the presence of more medium, medium-high, high, and very high ratings (high risk) than low and low-medium ratings (low risk) on 12 risk categories. A Brief Risk Interview score greater than 1 was used in determining whether or not to prescribe patients opioids.

^c^ The total study sample was 397 patients, but only 155 of the total participants had urine drug screening information available. Moreover, only those patients who were suspected of misusing opioids underwent urine drug screening.

Other risk assessment tools we evaluated, several of which are in common use, addressed traditional risk factors (such as personal and family history of substance use disorder or mental health disorder or diagnosis). These included the Opioid Risk Tool (LR, 1.5; 95% CI, 0.76-2.9), Brief Risk Questionnaire (LR, 1.2; 95% CI, 0.96-1.6), Brief Risk Interview (LR, 1.2; 95% CI, 0.96-1.6), and Screener and Opioid Assessment for Patients with Pain (LR, 1.2; 95% CI, 0.94-1.4) (tools’ characteristics are shown in Table 3 and eTable 2 in the Supplement).^18,19^ Furthermore, when their ability to discern patients at high vs low risk was critically assessed using 95% confidence intervals of LRs, none of these risk assessment tools appeared useful (Table 2).

**Table 3.** Characteristics of Opioid Risk Assessment Tools From High Quality Studies Included in Quantitative Synthesis

Instrument^a^	Source	Items, No.	Scope	Response Format	Before or During Opioid Therapy	Score Range	Usual Cutpoint	Literacy Level	Administration or Completion Time, min	Quality Score
Prescription Monitoring Questionnaire	Jones, et al,^18^ 2015	26	Specific to prescription opioids in chronic pain care	0 = Never/disagree to 4 = ≥4 times/agree	During	0-104	<20.5: Low risk; 20.5-30.0: moderate; 33.3-66.7: high	Easy	Approximately 10	III
Opioid Risk Tool	Jones et al,^18^ 2015	10	Specific to prescription opioids	Yes or no	Before	0-26	0-3: Low; 4-7: moderate; ≥8: high	Easy	<1	III
Brief Risk Questionnaire	Jones et al,^18^ 2015	12	Specific to prescription opioids for chronic pain	Yes or no and rating scales	During	0-24	≥3	Easy	Unclear	III
Brief Risk Interview	Jones et al,^18^ 2015	12	Specific to prescription opioids for chronic pain	Rating scales from low to very high risk	During	NA	≥1 Area with the highest risk rating	Easy	6-12	III
Screener and Opioid Assessment for Patients with Pain	Akbik et al,^19^ 2006	14	Specific to prescription opioids in chronic pain care	0 = Never to 4 = very often	Before	0-56	≥8	Easy	<8	III

Abbreviation: NA, not applicable.

^a^ See eTable 2 in the Supplement for full list and description of all available screening tools.

## Discussion

Recently published estimates suggest that more than one-third of US civilian, noninstitutionalized adults used prescription opioids in 2015.^1^ However, despite the public health emergency related to prescription opioid addiction, this review demonstrated that there are few quality studies available to help health care professionals determine which patients are likely to develop OUD and that incidence of prescription opioid addiction has likely been underestimated in high-quality (level ≥3) studies.^18,19^ Among single high-quality studies, having a history of a substance use disorder (opioid or nonopioid) or mental health diagnosis (eg, personality disorder, somatoform disorder, psychotic disorder, or anxiety disorder) was associated with a substantial increase in the likelihood of developing a prescription OUD. Furthermore, certain opioid prescription characteristics, including a duration of 30 days or greater, a daily dose greater than 120 morphine milligram equivalents, and concurrent prescriptions of atypical antipsychotics, were associated with an increase in the likelihood of developing a prescription OUD. However, few of these findings have been replicated in more than 1 study. Quality replication studies are needed. Furthermore, only the absence of mood disorder produced negative LRs that modestly lowered risk for prescription OUD, whereas none of the other findings were useful for identifying patients at lower risk. Finally, despite risk assessment tools becoming a widespread aspect of the prescribing of opioids in pain management care environments,^22^ they appeared to be of little value for identifying patients at high vs low risk.

While not relevant to the assessments of most individual characteristics or medication characteristics, one explanation for the poor performance of risk prediction tools may be that the act of screening may change patient behavior and/or decision making by physicians, lowering the overall incidence rate of prescription OUD.^23^ In other words, risk prediction tools may have low apparent diagnostic accuracy because the assessment process itself lowers the incidence of prescription OUD in patients at high risk. For instance, the PMQ has a series of questions that can be used to discuss the dangers of opioid addiction, which may cause the tool itself to increase patient awareness and encourage patients to limit opioid use. This may not be relevant to high-quality studies in which risk assessment and prescribing behavior were independent.^20,21^ While this explanation requires further investigation, from an evidence-based medicine perspective, the findings of this review nevertheless suggest that health care professionals do not have good tools to identify patients at high risk for developing prescription OUD or those at low risk, for whom prescription opioids can be safely prescribed.

While these findings are disheartening given the urgent need to identify more effective strategies to manage patients with chronic pain, they are well aligned with the evolving literature regarding risk of prescription OUD among individuals prescribed opioid medications for pain management, and the discordance between this literature and common prescribing behaviors in North America. For instance, while recent reports have demonstrated that physician perception of the low risk of prescription opioids may be based on flawed or limited studies,^24^ more rigorous reviews have highlighted the risks of prescription opioids and the limited evidence of benefit.^25,26^ For instance, the systematic review for the Pathways to Prevention Workshop, which was sponsored by the US National Institutes of Health, highlighted questions about the clinical effectiveness of prescription opioids in the chronic pain context and reported a dose-dependent risk for serious harms of long-term opioid therapy for chronic pain.^27^ More recently, the Strategies for Prescribing Analgesics Comparative Effectiveness (SPACE) trial found that treatment with prescription opioids was not superior to treatment with nonopioid medications on pain-related function in patients with chronic back pain or hip or knee osteoarthritis pain.^28^ Together with the lack of diagnostic value of the tools in widespread use for identifying patients at high or low risk, as identified in our review, this evolving literature on the limitations and harms of prescription opioids highlights the need to reconsider the common prescription of opioids for minor acute pain conditions and chronic pain.^25,26^

### Limitations

This study has limitations. While prior reviews have attempted to describe risk factors or opioid risk screening tools that can be used to classify patients into high- vs low-risk categories, to our knowledge, none have conducted rigorous quality assessments or used LRs as a strategy to assess the diagnostic utility of screening for risk factors or screening tools.^16,22,29,30,31^ Recently, an earlier similar review^16^ on this topic has been updated,^32,33^ but our review used a more systematic approach and quality assessment of reviewed studies. Specifically, our study quality assessment resulted in the exclusion of most studies included in earlier reviews on this topic.^32,33^ Nevertheless, the present review’s limitations highlight the gaps that exist in the literature in this area (eg, the identified heterogeneity among the included studies due to patient population, care setting, and criteria for diagnosis). In fact, the diagnosis of prescription opioid addiction in the context of chronic pain can be challenging,^34^ and studies included in this review used varying definitions (eg, *DSM-III*, *DSM-IV*, *ICD-9*) for the diagnosis of prescription opioid addiction. Because its definition has not been consistent in the pain literature, we considered a wide definition of prescription opioid addiction based on the definitions used by study authors (eTable 8 in the Supplement). Although the low incidence of OUD found in the large cohort studies^20,21^ might suggest that patients with an OUD diagnosis in those studies were more likely to have severe OUD, this explanation requires further investigation.

While it would be hoped that individual patient characteristics and risk screening tools might prove useful, when subjected to critical review, only a few individual patient characteristics (often identified in only 1 unreplicated study) helped identify patients at high risk, and no individual patient characteristics, other than the absence of a mood disorder, or screening tools appeared particularly useful for safely identifying patients at lower risk. Instead, most screening tools, including the commonly used Opioid Risk Tool,^35,36^ were based on lower-quality studies or, when test performance was assessed by calculating LRs, demonstrated poor diagnostic performance.

## Conclusions

This review found that a history of opioid or nonopioid substance use disorder, concomitant prescription of certain psychiatric medications, prolonged duration of opioid prescriptions (≥30 days), higher daily opioid doses, and a history of certain mental health disorders may be useful for identifying patients at high risk for prescription OUD, whereas only the absence of a mood disorder was useful for identifying patients at lower risk. The review also found that most screening tools are from low-quality studies and that no screening tool was particularly useful for identifying patients for whom opioids can be safely prescribed. Furthermore, few high-quality studies exist that can inform clinicians and policy makers on how to manage this difficult and important public health problem. These findings, alongside persistently high rates of opioid prescribing and the literature demonstrating the overall harms and limited benefits of prescription opioids in many pain conditions,^25,26,27,28^ suggest that prescribers should be better aware of the major diagnostic limitations of assessing patient characteristics and using screening tools when seeking to identify patients who may safely be prescribed opioid medications.

## Notes

Supplement. **eAppendix.** Search Methods and Search Strategy **eFigure.** Flowchart of Studies **eTable 1.** Features of Included Studies **eTable 2.** Opioid Risk Assessment Tools **eTable 3.** QUADAS Assessment of Included Articles Applied to Prescription Opioid Addiction Risk **eTable 4.** QUADAS Tool Results **eTable 5.** Results From Individual Studies—Variables Reported in 2 High-Quality Studies **eTable 6.** Results From Individual Studies—Variables Reported Only in 1 High-Quality Study **eTable 7.** Risk Factors That Predict Prescription Opioid Use Disorder Among Opioid Naïve Patients Initiating Prescription Opioids **eTable 8.** Clinical Criterion Standards for Opioid Use Disorder in Pain Management Among the Studies Included in the Review **eReferences**
