# Supplementary material for: Strategies to Identify Patient Risks of Prescription Opioid Addiction When Initiating Opioids for Pain: A Systematic Review
Source: JAMA Netw Open. 2019 May 3;2(5):e193365. doi: 10.1001/jamanetworkopen.2019.3365 (PMC6503484; doi:10.1001/jamanetworkopen.2019.3365)
Supplement: Supplement. — eAppendix. Search Methods and Search Strategy eFigure. Flowchart of Studies eTable 1. Features of Included Studies eTable 2. Opioid Risk Assessment Tools eTable 3. QUADAS Assessment of Included Articles Applied to Prescription Opioid Addiction Risk eTable 4. QUADAS Tool Results eTable 5. Results From Individual Studies—Variables Reported in 2 High-Quality Studies eTable 6. Results From Individual Studies—Variables Reported Only in 1 High-Quality Study eTable 7. Risk Factors That Predict Prescription Opioid Use Disorder Among Opioid Naïve Patients Initiating Prescription Opioids eTable 8. Clinical Criterion Standards for Opioid Use Disorder in Pain Management Among the Studies Included in the Review eReferences [file jamanetwopen-2-e193365-s001.pdf]

Supplementary Online Content

Klimas J, Gorfinkel L, Fairbairn N, et al. Strategies to identify patient risks of prescription opioid addiction when initiating opioids for pain: a systematic review. *JAMA Netw Open*. 2019;2(5):e193365. doi:10.1001/jamanetworkopen.2019.3365

eAppendix. Search Methods and Search Strategy
eFigure. Flowchart of Studies
eTable 1. Features of Included Studies
eTable 2. Opioid Risk Assessment Tools
eTable 3. QUADAS Assessment of Included Articles Applied to Prescription Opioid Addiction Risk
eTable 4. QUADAS Tool Results
eTable 5. Results From Individual Studies—Variables Reported in 2 High-Quality Studies
eTable 6. Results From Individual Studies—Variables Reported Only in 1 High-Quality Study
eTable 7. Risk Factors That Predict Prescription Opioid Use Disorder Among Opioid Naïve Patients Initiating Prescription Opioids
eTable 8. Clinical Criterion Standards for Opioid Use Disorder in Pain Management Among the Studies Included in the Review
eReferences

This supplementary material has been provided by the authors to give readers additional information about their work.

eAppendix. Search methods and search strategy

Eligible studies compared patient symptoms and signs among patients being newly prescribed opioids for pain who did or did not subsequently develop prescription OUD. Studies assessing screening tools that utilized combinations of symptoms and signs were also eligible. To identify relevant articles, MEDLINE and EMBASE from January 1946 to October 2017 were searched. Search strategy terms included *opioid-related disorders*, MESH terms *substance related disorders*, *pain*, *analgesics*, and terms previously found to be useful for retrieving diagnostic studies (see Search Strategy).[1] Additional studies were identified by searching reference lists of original studies and review articles.

Study Selection

Two reviewers (LG and JK) independently screened abstracts for inclusion. Studies that evaluated prescription characteristics, patient characteristics, past substance use disorders, mental health disorders and screening tools assessing the risk of prescription opioid addiction in the context of pain management were included. Articles not reporting original data (i.e. review articles) were also excluded. To be eligible for the present review, we also restricted to studies of opioid naïve patients newly starting opioid medications for pain and excluded studies assessing for a diagnosis of OUD among patients already on opioid-based medications.

Outcome measures

The following outcomes were assessed: symptoms, signs, risk factors, and scores on screening tools of patients who subsequently did and did not develop prescription OUD. As there is currently no gold standard for the diagnosis of OUD in pain patients that has been described in the literature,[2] and since the diagnostic criteria for OUD have evolved over time.[3] We allowed for the definitions that have been used in the literature including a diagnosis of OUD using the Diagnostic and Statistical Manual (DSM), and diagnoses of opioid "abuse" and "dependence" using the DSM-III, DSM–IV, ICD-9, or ICD-10. In addition, we included eligible studies where the presence of aberrant drug-related behaviors and failed urine drug screens was taken as a valid proxy for the above in articles of diagnostic screening in pain care.

Data extraction

All citations identified by searches were independently screened based on title and abstract by two reviewers (LG, JK). Each potentially relevant study was then reviewed in full text and assessed for all inclusion criteria. Any disagreements were resolved by discussion among reviewers and senior authors (JK, EW). Relevant data from eligible articles (i.e., patient and treatment characteristics, outcomes, etc.) were then extracted.

Quality Assessment

Two reviewers (LG and LA/JK) rated study quality using a five-level Hierarchy of Evidence rating scale by Simel and Rennie used as part of the *Journal of the American Medical Association*'s Rational Clinical Examination series (2008).[4] Using this schema, Level 1 indicated the highest quality and was assigned to studies that had independent blinded comparison of the symptoms or signs with a valid criterion standard in a large number of consecutive patients (for this review defined as greater than 150).[4] Level 2 studies were similar to level 1 studies but enrolled fewer than 150 patients. Level 3 studies enrolled nonconsecutive patients. Level 4 studies used non-independent comparisons among a "convenience" sample of patients at risk of having the prescription OUD. Consistent with prior reviews in this series,[4] only studies that met the quality standards of Level 1, 2, or 3 were included. In accordance with the recommendations of the Preferred Reporting Items for Systematic Reviews and Meta-Analyses (PRISMA-DTA), and Standards for Reporting Diagnostic Accuracy (STARD), sources of bias were also evaluated with the Quality Assessment of Diagnostic Accuracy Studies (QUADAS) Tool.[5-8]

Data Synthesis and Analysis

The population incidence of prescription OUD after opioid prescription was estimated by collating data on opioid "dependence" and "abuse" from reports of the Cochrane Collaboration and from previous reviews on the topic.[9-11] In brief, data on the incidence of prescription OUD in opioid-naïve patients being prescribed opioids for pain was extracted from the studies that met the eligibility requirement for this review. Here, summary incidence was calculated using a random effects estimate from the included studies and performed via a Comprehensive Meta-analysis (version 3) software.[12] Contingency tables (2x2) were constructed to estimate the likelihood ratios (LR), sensitivity, and specificity for each risk factor or screening tool. Data were entered into Microsoft Excel spreadsheets predesigned to calculate the sensitivity, specificity, LRs, and their 95% CIs.

When a symptom, sign or risk factor was assessed in only one high quality study, the LR and 95% confidence interval (CI) were reported. When a symptom, sign or risk factor was assessed in two studies, the range of LRs was reported. If a symptom, sign or risk factor was considered in three or more studies, the protocol sought to pool the LR data using separate univariate random-effects meta-analysis.

Search strategy

Prescription Opioid Addiction Risk Searches Novemer 1, 2018

MEDLINE 1946 to November 2018

1	physical exam*.mp. or exp Physical Examination/	1327458
2	(sign* or symptom*).mp.	7234025
3	exp Medical History Taking/	20334
4	risk factor*.mp.	1006422
5	(age* or gender* or sex* or residen* or income*).mp.	11263471
6	exp Professional Competence/	107195
7	or/1-6	15219319
8	exp "Reproducibility of Results"/	366279
9	reproducib*.mp.	471203
10	exp Observer Variation/	39838
11	exp Diagnostic Tests, Routine/	10249
12	exp Decision Support Techniques/	72030
13	exp Bayes Theorem/	29060
14	or/8-13	589656
15	(buprenorphine or dihydromorphine or diamorphine or hydromorphone or methadone or morphine or opioid* or opiate* or oxycodone or fentanyl or levorphanol or pethidine or meperidine).mp.	171253
16	exp Analgesics, Opioid/	105976
17	(substance adj3 disorder*).mp. or exp Substance-Related Disorders/	267324
18	(opioid adj3 disorder*).mp. or exp opioid-related disorders/	24121
19	exp Opioid-Related Disorders/ or exp Methadone/ or exp Analgesics, Opioid Dependence/	29228
20	(abuse* or abusing or addict* or misuse or dependen* or disorder* or withdrawal* or abstinen* or abstain* or detox*).mp.	3557014
21	or/15-20	3734502
22	(pain or painful).mp. or exp pain/	746603
23	7 and 14 and 21 and 22	2953
24	(sensitivity and specificity).mp.	455480
25	exp "Sensitivity and Specificity"/	537195
26	24 or 25	642667
**27**	**23 and 26**	635

EMBASE 1974 to November 2018

1	physical examination.mp. or exp Physical Examination/	261510
2	(sign* or symptom*).mp.	9567284
3	exp anamnesis/	208871
4	risk factor*.mp.	1184188
5	(age* or gender* or sex* or residen* or income*).mp.	9826947
6	exp Professional Competence/	30328
7	or/1-6	15633278
8	exp “Reproducibility of Results”/	196585
9	reproducib*.mp.	312261
10	exp observer variation/	19215
11	exp diagnostic test/	827952
12	exp decision support system/	21362
13	exp Bayes theorem/	30203
14	or/8-13	1186699
15	(buprenorphine or dihydromorphine or diamorphine or hydromorphone or methadone or morphine or opioid* or opiate* or oxycodone or fentanyl or levorphanol or pethidine or meperidine).mp.	312464
16	exp narcotic analgesic agent/	301389
17	(substance adj3 disorder*).mp. or exp drug dependence/	224240
18	(opioid adj3 disorder*).mp. or exp opiate addiction/	17276
19	exp methadone/ or exp narcotic analgesic agent/	39793
20	(abuse* or abusing or addict* or misuse or dependen* or disorder* or withdrawal* or abstinen* or abstain* or detox*).mp.	4778043
21	15 or 16 or 17 or 18 or 19 or 20	5057063
22	(pain or painful).mp. or exp pain/	1441095
23	7 and 14 and 21 and 22	21622
24	(sensitivity and specificity).mp.	442555
25	exp “sensitivity and specificity”/	307360
26	24 or 25	442555
**27**	**23 and 26**	559
**TOT**	**Combined EMBASE and Medline search**	1194

eFigure. Flowchart of studies

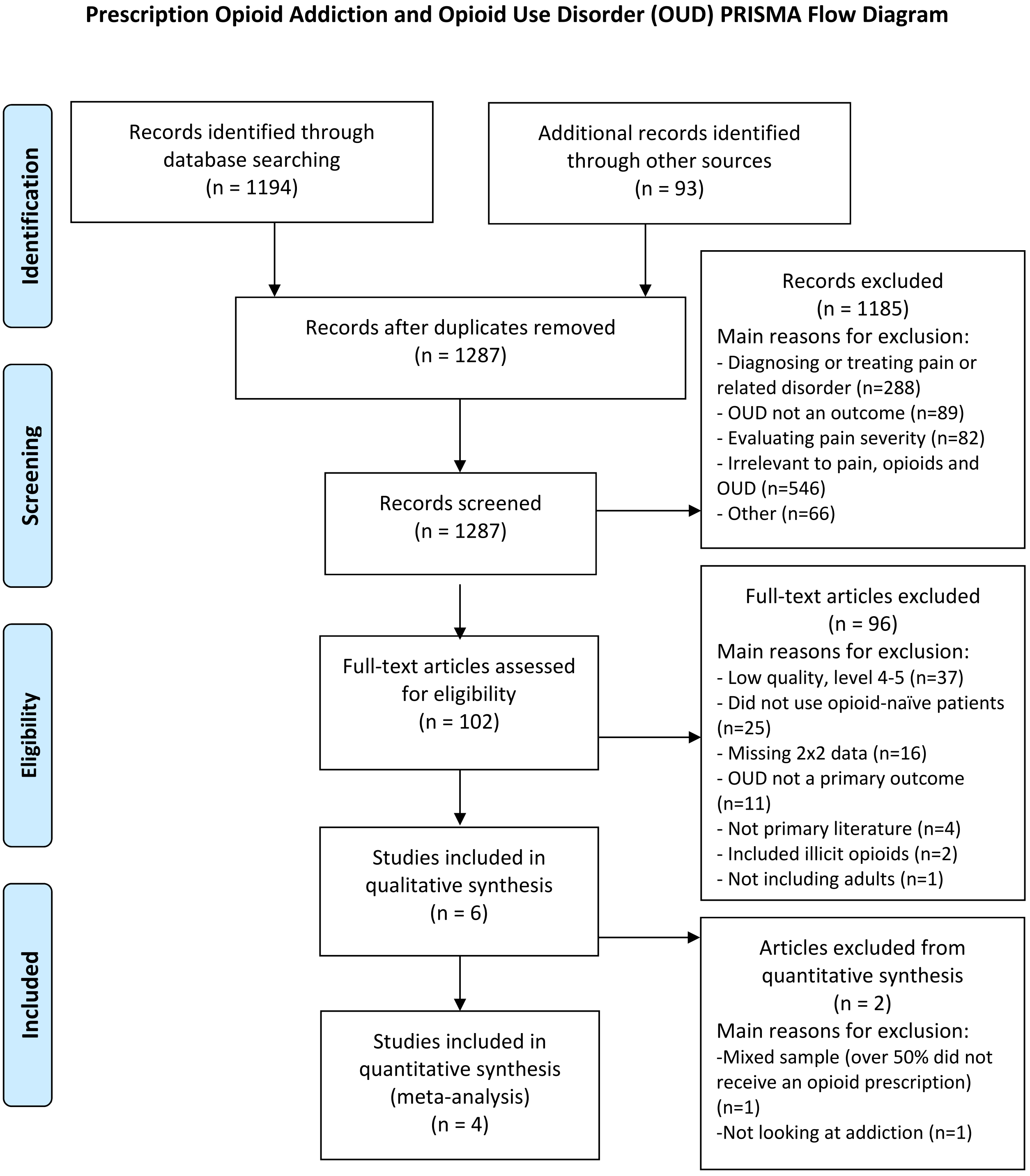

eTable 1. Features of Included Studies

Study	Quality assessment	Sample size	Study design	Study location	No. Prescription OUD (%)	Study population	Findings reported
**Studies included in quantitative synthesis**							
Akbick et al. 2006	III	397	Prospective observational study	U.S.A. (city unspecified)	44 (11.1%)*<br><br>*based on UDS for illicit drugs alone	238 patients prescribed opioids for pain at a tertiary hospital, 159 patients prescribed opioids for pain at a Veterans Administration Pain Center	SOAPP compared to urine drug screen, race, gender, age
Cochran et al. 2014	I	2,841,793	Retrospective observational study using a medical insurance database	U.S.A.	2,913 (0.102%)	Patients in a nation-wide medical insurance database	Prescription OUD development compared to gender, region, marriage status, period substance use, concurrent mental disorders, concurrent medications, age, opioid characteristics, hospital visits
Edlund et al. 2010	I	46,256	Retrospective observational study using a commercial insurance database	U.S.A.	1,465 (3.17%)	Commercially-insured patients on 1 of 2 insurance databases who received opioid therapy for at least 90 days following prescription index date	Prescription OUD development compared to age, gender, pain type, mental disorders, prior substance use, prescription opioid characteristics
Jones et al. 2015	III	142	Prospective cohort study	Tennessee, Knoxville USA	48 (33.8%)	New patients being considered for a trial of opioids for a chronic pain condition in a psychology practice	Predictive ability of ORT, BRQ, BRI, and PMQ for aberrant drug-related behavior
**Studies excluded from quantitative synthesis**							
Clarke et al. 2014	III	19,256	Retrospective observational study	Ontario, Canada	1229 (6.38%)	Opioid-naive Ontario residents who were aged 66 years or older and underwent any one of nine prespecified elective major surgical procedures	Prolonged opioid use compared to age, gender, income, surgical procedure, comorbid disease, preoperative drugs

Study	Quality assessment	Sample size	Study design	Study location	No. Prescription OUD (%)	Study population	Findings reported
Hooten et al. 2015	I	293	Retrospective observational study	Rochester, Minnesota	19 (6.48%)	Patients receiving an opioid prescription from one of two medical centers	Chronic opioid use compared to age, gender, race, education, psychiatric history, cause of pain, substance use history

*Total N = 397, but only 155/397 of the total participants had Urine Drug Screening information available. Moreover, only those patients who were suspected of "misusing" opioids underwent urine drug screening.

eTable 2. Opioid risk assessment tools

Instrument	Study (inclusion/ reason for exclusion)	No. of Items	Administered by	Scope	Response Format	Before or during opioid therapy	Score Range	Usual Cutpoint	Literacy Level	Administration or Completion Time, min
Addiction Behavior Checklist (ABC)	Wu 2006[13] (not incidence)	20	Patient Interview	Specific to prescribed opioids or sedative analgesics	Yes or No	During	0-20	≥3	average	~10 min
Chabal 5-Point Prescription Opioid Abuse Checklist	Chabal 1997[14] (not incidence)	5	Completed by healthcare provider	Specific to prescription opioids	Yes or No	During	0-5	≥3	n/a	<1 min
Current Opioid Misuse Measure (COMM)	Meltzer 2011[15] (QL= 4-5) Butler 2007[16] (not incidence) Butler 2010[17] (not incidence)	17	Patient interview	Specific to prescription opioids	"0=Never" to "4=Very often"	During	0-68	≥9	easy	<10 min
Opioid Risk Tool (ORT)	Witkin 2013[18] (QL = 4-5) Webster 2005[19] (QL = 4-5) Jones 2015[20] (included)*	10	Patient interview	Specific to prescription opioids	Yes or No	Before	0-26	0-3: low 4-7: moderate ≥8: high	easy	<1 min
Pain Assessment and Documentation Tool (PADT)	Passik 2004[21] (Participants were not patients)	41	Completed by healthcare provider(s)	Overall opioid effects with misuse category	Yes or No	During	No numerical scoring method	n/a	n/a	2-5 min
Pain Medication Questionnaire (PMQ)	Dowling 2007[22] (QL= 4-5) Højsted 2011[23] (Includes cancer pain) Buelow 2009[24] (not incidence) Holmes 2006[25] (not incidence) Adams 2004 [26] (not incidence) Jones 2015[20] (included)*	26	Patient self-complete	Specific to prescription opioids in chronic pain care	0="Never"/ "Disagree" to 4="4+ times" / "Agree"	During	0-104	<20.5: low risk 20.5-30.0: moderate 33.3-66.7: high	easy	~10 min
Prescribed Opioid Difficulties Scale (PODS)*	Banta-Green 2010[27] (QL= 4-5)	15	Patient self-complete	Overall difficulties with chronic pain opioid therapy	"Strongly Disagree=0" to "Strongly Agree=4"	During	0-61	8-15: medium 16+: high	average	25-30 min
Prescription Drug Use Questionnaire (PDUQ)	Compton 1998[28] (QL = 4-5)	42	Patient interview	Specific to prescription opioids in chronic pain	Yes or No	During	0-42	≥11	average	~20 min
Prescription Drug Use Questionnaire – patient version (PDUQp)	Compton 2008[29] (not incidence)	42	Patient self-complete	Specific to prescription opioids in chronic pain	Yes or No	During	0-42	≥10	average	~20 min

Prescription Drug Use Questionnaire – psychiatric subscale	Wasan 2007[30] (not incidence)	5	Patient interview	Specific to prescription opioids in chronic pain	Yes or No	During	0-5	>1	average	<1 min
Prescription Opioid Misuse Index (POMI)	Knisely 2008[31] (QL = 4-5)	9	Patient interview	Specific to prescription opioids	Yes or No	During	0-9	≥2	easy	<5 min
Screener and Opioid Assessment for Patients with Pain (SOAPP)	Butler 2004[32] (QL = 4-5) Akbick 2006[33] (included)*	14	Patient interview	Specific to prescription opioids in chronic pain care	0="Never" to 4="Very Often"	Before	0-56	≥7	easy	<8 min
Revised Screener and Opioid Assessment for Patients With Pain (SOAPP-R)	Brown 2011[34] (QL = 4-5) Butler 2009[35] (not incidence) Butler 2008[36] (not incidence)	24	Patient self-complete, observation and toxicology by healthcare professional	Specific to prescription opioids in pain care	1="not at all important" to 5= "very important"	Before	24-120	≥18	easy	~5 min
The Diagnosis, Intractability, Risk, Efficacy (DIRE) tool	Belgrade 2006[37] (QL = 4-5)	7	Completed by healthcare provider	Specific to prescription opioids in chronic pain care	1 to 3 based on question-specific explanations	Before	7-21	7-13: low risk 14-21: high risk	n/a	<2 min
Screening Instrument for Substance Abuse Potential (SISAP)	Coambs 1996[38] (QL = 4-5)	5	Patient interview	Specific to prescription opioids in pain management	Yes or No based on question-specific explanations	Before	0-5	3	easy	<1 min
Screening Tool for Abuse (Atluri tool)	Atluri 2004[39] (QL = 4-5)	6	Completed by healthcare provider	Specific to prescription opioids	Yes or No	During	0-6	≥3	n/a	unclear
Temple STAR questionnaire	Friedman 2003[40] (QL = 4)	11	Patient self-complete	Specific to prescription opioids in chronic pain care	Yes or No	During	0-11	unclear	easy	unclear
CAGE Adapted to Include Drugs (CAGE-AID)	Not yet tested on pain patients	4	Patient interview or self-report	For alcohol and all drugs	Yes or No	During	0-4	≥3	easy	~1 min
The Proove Opioid Risk (POR) Algorithm	Brenton 2017[41] (QL = 4-5)	11 genetic markers and 5 clinical factors	Genetic testing and patient self-complete	For all opioids	Yes or No	Before	unclear	1-11: low 12-23: moderate ≥24: high risk	n/a	unclear
Addiction Risk Questionnaire (ARQ)	Not yet validated, tool proposed by Leonardi 2015[42]	28	Patient interview or self-complete	Specific to general practitioners and prescription opioids in chronic pain	Yes or No and "1=Totally agree" to "4=Strongly disagree"	Before	None (not yet validated)	None (not yet validated)	easy	unclear
Opioid-Related Behaviours in Treatment (ORBIT) scale	Larance 2016[43] (QL = 4-5)	10	Patient self-complete	Specific to long-term opioid therapy	"0=Never" to "4=Very often"	During	0-40	None (not yet validated)	easy	unclear
The Brief Risk Questionnaire (BRQ)	Jones 2015[20] (included)*	12	Patient self-complete	Specific to prescription opioids for chronic pain	Yes or No and Rating Scales	During	0-24	≥3	easy	unclear
The Brief Risk Interview (BRI)	Jones 2013[44] (QL = 4-5) Jones 2014[45] (QL = 4-5)	12	Patient interview	Specific to prescription opioids for chronic pain	Rating Scales from low- to very high risk	During	n/a	At least 1 area with the highest	easy	6-12 min

	Jones 2015[20] (included)*							risk rating		
Opioid Abuse Risk Screener (OARS)	Averill 2017[46] (no 2x2 data)	38 or 43 (multiple versions)	Patient self-complete	Specific to prescription opioids	0=strongly disagree 3=strongly agree	Before	0-84	unclear	un-available	unclear
Fleming 12 Aberrant Drug Related Behaviors Checklist	Fleming 2008[47] (not incidence)	12	Patient self-complete	Specific to prescription opioids for chronic pain	"0=Never" to "4=Four or more times"	During	0-48	≥9	average	unclear
Manchikanti unnamed illicit drug screener	Manchikanti 2003[48] (QL = 4-5) Manchikanti 2004[49] (not incidence)	4, 8, or 12 (multiple versions)	Completed by healthcare provider	Specific to prescription opioids for chronic pain	Yes or No	During	0-4, 0-8, or 0-12	≥2 on items 3, 4, 5 and 7	n/a	unclear
Opioid Compliance Checklist (OCC)	Jamison 2016[50] (no 2x2 data) Jamison 2014[51] (not incidence)	5 or 8 (multiple versions)	Patient self-complete	Specific to prescription opioids in chronic pain care	Yes or No	During	0-5	≥1	average	unclear
Patient Opioid Therapy Questionnaire (POTQ)	Michna 2004[52] (not incidence)	3[§]	Patient interview	Specific to prescription opioids in chronic non-cancer pain	Yes or No	During	0-3	0-1: low risk 2-3 high risk	n/a	unclear
Portenoy's Criteria	Højsted 2010[2] (not incidence)	10	Patient self-report	Specific to prescription opioids in chronic non-cancer pain	Yes or No	During	0-10	Positive responses to first 2 items, plus at least 1 positive response on the next 8 items	average	unclear
Opioid-related Overdose Risk Behavior Scale (ORBS)	Pouget 2017[53] (not looking at medically prescribed opioids)	25	Patient Interview	Specific to prescription opioids	Yes or No	During	0-25	unclear	easy	5-10 min
Overdose Risk InfOrmatioN (ORION) tool	Carra 2017[54] (QL = 4-5)	9 risk factors	Online software for clinician use	For estimating overdose risk in the context of any OUD	Yes or No	During	0-100	Results presented on a continuum (0=lowest risk, 100=highest risk)	easy	~5 min

*High quality studies included in the current review; QL= quality level according to the JAMA Rational Clinical Examination (RCE) quality assessment (lowest quality=level 5, highest quality=level 1). Studies with quality levels 4-5 were excluded from this review.

§Previous studies, including Butler et al. 2007, Butler et al. 2008, Butler et al. 2009, and Butler et al. 2010, have reported using an 11-item version of the POTQ scale involving physician ratings. We were unable to identify a validation study for this version of the POTQ, and such a scale appears unmentioned in the original cited study (Michna 2004).

eTable 3. QUADAS Assessment of Included articles applied to prescription opioid addiction risk

1. Was the spectrum of patients representative of the patients who will receive the test in practice? *Patients at risk of opioid addiction (condition) = yes. If no risk of OUD = no.*

2. Were selection criteria clearly described? *If reproducible = yes.*

3. Is the reference standard likely to correctly classify the OUD? *If standard laboratory techniques used to diagnose opioid addiction = yes. If ambiguous = no.*

4. Is the time period between reference standard and index test short enough to be reasonably sure that the target condition did not change between the two tests? *If OUD testing and assessment done as part of the same consultation or research study site visit = yes. If reported duration between assessment and OUD testing more than 2 days = no.*

5. Did the whole sample or a random selection of the sample, receive verification using a reference standard of diagnosis? *Yes or no.*

6. Did patients receive the same reference standard regardless of the index test result? *Yes or no.*

7. Was the reference standard independent of the index test (i.e. the index test did not form part of the reference standard)? *Yes or no.*

8. Was the execution of the index test described in sufficient detail to permit replication of the test? *If description adequately described to allow for replication, including a symptom definition, = yes.*

9. Was the execution of the reference standard described in sufficient detail to permit its replication? *If laboratory approach to diagnosing OUD described then = yes.*

10. Were the index test results interpreted without knowledge of the results of the reference standard?

11. Were the reference standard results interpreted without knowledge of the results of the index test?

12. Were the same clinical data available when test results were interpreted as would be available when the test is used in practice? *When the test executer had as much info as in clinical practice = yes.*

13. Were uninterpretable/ intermediate test results reported? *Not reported, numbers are correct = yes*

14. Were withdrawals from the study explained? *Not reported, numbers are correct = yes*

a1. Did the study provide a clear definition of what was considered to be a ‘positive’ result?**

a2. Was treatment withheld until both the index test and reference standard were performed?**

*all items are scored yes, no or unclear
**additional QUADAS tool item

eTable 4. QUADAS tool results (see eTable 2 for QUADAS tool items)

	QUADAS tool items															
Author, year of publication	1	2	3	4	5	6	7	8	9	10	11	12	13	14	a1	a2
**Studies included in quantitative synthesis**																
Akbik et al, 2006[33]	Y	N	Y	U	N	Y	Y	Y	Y	U	Y	Y	Y	Y	Y	U
Cochran et al, 2014[55]	U	Y	Y	n/a	Y	n/a	n/a	n/a	Y	n/a	n/a	U	Y	Y	Y	n/a
Edlund et al, 2010[56]	Y	Y	Y	n/a	Y	Y	n/a	n/a	U	n/a	n/a	Y	Y	N	Y	n/a
Jones et al, 2015[20]	Y	N	N	n/a	Y	Y	N	Y	Y	Y	Y	Y	Y	Y	Y	U
**Studies excluded from quantitative synthesis**																
Clarke et al, 2014[57]	N	Y	N	n/a	Y	n/a	n/a	n/a	Y	n/a	n/a	Y	Y	Y	Y	n/a
Hooten et al, 2015[58]	Y	Y	N	n/a	Y	n/a	n/a	n/a	Y	n/a	n/a	Y	Y	Y	Y	U

n/a indicates a study in which there was no index test. These were retrospective cohort studies that looked at the characteristics of patients that did vs did not develop OUD following an opioid prescription.

eTable 5. Results from individual studies – variables reported in 2 high-quality studies

Finding	Reference #	No. with finding / sample size (%)	Sensitivity (95% CI)	Specificity (95% CI)	LR+ (95% CI)	LR- (95% CI)
**Symptoms and features on patient history**						
**Gender (male)**	Edlund et al, 2010[56]	17746 / 46256 (38%)	0.33-0.41 (range)*	0.62-0.72 (range)	1.1-1.2 (range)	0.94-0.96 (range)
	Cochran et al, 2014[55]	1255458 / 2,841,793 (44%)	0.60 (0.58-0.62)	0.56 (0.56-0.56)	1.4 (1.3 – 1.4)	0.72 (0.69 – 0.75)
**Past SUD (non-opioid)**	Edlund et al, 2010[56]	1375 / 46256 (3.0%)	0.14-0.23 (range)*	0.95-0.98 (range)	4.2-7.7 (range)	0.82-0.88 (range)
	Cochran et al, 2014[55]	98220 / 2841793 (3.5%)	0.58 (0.56-0.59)	0.97 (0.97-0.97)	17 (16 – 18)	0.44 (0.42 – 0.46)

Abbreviations: LR = Likelihood Ratio; CI = Confidence Interval. LR calculated directly from 2x2 tables and then rounded. SUD = substance use disorder. * The LR range is derived from two separate databases described in this study.[56]

eTable 6. Results from individual studies – variables reported only in 1 high-quality study

Finding	Reference #	No. with finding / sample size (%)	Sensitivity (95% CI)	Specificity (95% CI)	LR+ (95% CI)	LR- (95% CI)
**Features of patient history**						
Condition under study: Opioid abuse or dependence						
**Any personality disorder**	55	848 / 2841793 (0.02%)	0.08 (0.05-0.12)	1.0 (1.0-1.0)	27 (18-41)	0.99 (0.99-1.0)
**Any pain disorder**	55	2913 / 2838880 (0.10%)	0.02 (0.02-0.03)	1.0 (1.0-1.0)	23 (18-29)	0.98 (0.98-0.99)
**Past opioid use disorder[a]**	56	1465 / 44791 (3.3%)	0.07-0.09 (range)	1.0-1.0 (range)	17-22 (range)	0.91-0.93 (range)
**Somatoform disorders**	55	1827 / 2841793 (0.06%)	0.08 (0.05-0.11)	1.0 (1.0-1.0)	12 (7.8-18)	0.99 (0.99-1.0)
**Psychotic disorders**	55	4986 / 2841793 (0.18%)	0.19 (0.15-0.25)	1.0 (1.0-1.0)	11 (8.5-14)	0.98 (0.98-0.99)
**Any mood disorder**	55	260963 / 2841793 (9.2%)	0.55 (0.53-0.56)	0.91 (0.91-0.91)	6.0 (5.8-6.2)	0.50 (0.45-0.52)
**Any anxiety disorder**	55	156952 / 2841793 (5.5%)	0.29 (0.27-0.31)	0.95 (0.95-0.95)	5.3 (5-5.6)	0.75 (0.74-0.77)
**2+ mental health disorders[a]**	56	277-1188/ 9651-36605 (2.9-3.3%)	...	...	2.8-5.3	...
**1 mental health disorder[a]**	56	277-1188 / 9651-36605 (2.9-3.3%)	...	...	1.3-1.9	...
**"0" mental health disorder[a]**	56	277-1188 / 9651-36605 (2.9-3.3%)	...	...	0.65-0.72	...
**Prescription characteristics**						
Condition under study: Opioid abuse or dependence						
**Concomitant medication: Atypical antipsychotic**	55	2913 / 2838880 (0.10%)	0.24 (0.22-0.25)	0.10 (0.10-0.10)	17 (15-18)	0.77 (0.76-0.79)

Finding	Reference #	No. with finding / sample size (%)	Sensitivity (95% CI)	Specificity (95% CI)	LR+ (95% CI)	LR- (95% CI)
**Concomitant medication: Anxiolytics** (Buspirone Hydrochloride)	55	2913 / 2838880 (0.10%)	0.08 (0.07-0.09)	0.99 (0.99-0.99)	7.3 (6.5-8.3)	0.93 (0.92-0.94)
**Concomitant medication: Tricyclics**	55	2913 / 2838880 (0.10%)	0.40 (0.38-0.06)	0.92 (0.92-0.92)	5.1 (4.8-5.3)	0.66 (0.64-0.68)
**Concomitant medication: Anticonvulsants**	55	2913 / 2838880 (0.10%)	0.34 (0.32-0.35)	0.93 (0.93-0.93)	5.0 (4.8-5.3)	0.71 (0.69-0.73)
**Concomitant medication: Other antidepressants**	55	2913 / 2838880 (0.10%)	0.45 (0.44-0.47)	0.88 (0.88-0.88)	3.8 (3.7-4.0)	0.62 (0.60-0.64)
**Concomitant medication: Benzodiazepines**	55	2913 / 2838880 (0.10%)	0.53 (0.51-0.54)	0.81 (0.81-0.81)	2.7 (2.6-2.8)	0.59 (0.58-0.61)
**Concomitant medication: Antipsychotics**	55	2913 / 2838880 (0.10%)	0.004 (0.002-0.007)	1.0 (1.0-1.0)	4.2 (2.4-7.3)	1.0 (1.0-1.0)
**Concomitant medication: SSRIs**	55	2913 / 2838880 (0.10%)	0.45 (0.43-0.47)	0.85 (0.85-0.85)	3.1 (2.9-3.2)	0.65 (0.63-0.67)
**Any opioid, all schedule types[a,#]**	56	1465 / 44791 (3.3%)	0.05-0.06 (range)	0.99-0.99 (range)	3.5-4.9 (range)	0.95-0.96 (range)
**Opioid dose >120 mg/day[a]**	56	1465 / 44791 (3.3%)	0.20-0.21 (range)	0.94-0.94 (range)	3.2-3.4 (range)	0.85-0.85 (range)
**Opioid type: Schedule II long and short-acting[a]**	56	1465 / 44791 (3.3%)	0.07-0.08 (range)	0.97-0.98 (range)	2.8-3.2 (range)	0.95-0.95 (range)
**Opioid type: Schedule II long-acting[a]**	56	1465 / 44791 (3.3%)	0.14-0.14 (range)	0.95-0.95 (range)	2.8-2.9 (range)	0.90-0.91 (range)
**Screening instruments**						
Condition under study: Aberrant drug-related behaviors						

Finding	Reference #	No. with finding / sample size (%)	Sensitivity (95% CI)	Specificity (95% CI)	LR+ (95% CI)	LR- (95% CI)
**Prescription medication questionnaire (PMQ) ≥ 30**	20	48 / 142 (34%)	0.35 (0.23-0.51)	0.86 (0.78-0.92)	2.6 (1.4-4.8)	0.75 (0.60-0.94)
**Opioid Risk Tools (ORT)[§] ≥ 4**	20	48 / 142 (34%)	0.25 (0.14-0.40)	0.83 (0.74-0.90)	1.5 (0.76-2.9)	0.90 (0.75-1.1)
**Brief Risk Questionnaire (BRQ) ≥ 3**	20	48 / 142 (34%)	0.73 (0.52-0.85)	0.40 (0.30-0.51)	1.2 (0.96-1.6)	0.67 (0.40-1.1)
**Brief Risk Interview (BRI)***	20	48 / 142 (34%)	0.69 (0.54-0.81)	0.45 (0.34-0.55)	1.2 (0.96-1.6)	0.70 (0.43-1.1)
Condition under study: Positive urine drug screen						
**Screener and Opioid Assessment for Patients with Pain (SOAPP) ≥ 8**	33	44 / 155 (28%)[¥]	0.59 (0.49-0.68)	0.48 (0.42-0.55)	1.2 (0.94-1.4)	0.85 (0.65-1.1)

[a]The LR range includes two disparate populations, 1) one national, commercially insured population (HealthCore in the West, Mid-West, and South-East regions of the U.S.) and 2) one state-based, publicly insured (Arkansas Medicaid serves "a disadvantaged and vulnerable population with the highest opioid use in the U.S.). Any mental health disorder was derived from the presence of adjustment disorder, anxiety disorder, mood disorder, personality disorder, and miscellaneous disorders (such as an eating disorder or somatoform disorder). For results on an ordinal scale (0, 1, 2 mental health disorders) the sensitivity, specificity, and LR- no longer apply. The LR represents the LR at increasing numbers of mental health disorders from 0 to ≥2.

*Positive test indicated by the presence of more 'medium', 'medium high' 'high' and 'very high' ratings (high risk) than 'low' and 'low medium' ratings (low risk) on 12 risk categories.

[§]Although this study[20] did not report high specificity (LR+), it is likely the most accessible of the reported tools as it can be accessed on a US government (.gov) website and has no copyright.

[#]Patients received at least 30 days supply of any opioid, i.e., Schedule III or IV AND short-acting schedule II AND long-acting schedule II opioids within a 6-month period.

[¥]Total N = 397, but only 155/397 of the total participants had Urine Drug Screening information available. Moreover, only those patients who were suspected of "misusing" opioids underwent urine drug screening.

eTable 7. Risk factors that predict Prescription Opioid Use Disorder among opioid naïve patients initiating prescription opioids.

Finding	Studies, Reference #	Sensitivity (95% CI)	Specificity (95% CI)	LR positive (95% CI)	LR negative (95% CI)
**Risk Factors**					
**Mental Health History**					
Any personality disorder	1[55]	0.08 (0.05-0.12)	1.0 (1.0-1.0)	27 (18-41)	0.99 (0.99-1.0)
Any pain disorder	1[55]	0.02 (0.02-0.03)	1.0 (1.0-1.0)	23 (18-29)	0.98 (0.98-0.99)
Past opioid use disorder (OUD)[a]	1[56]	0.07-0.09 (range)	1.0-1.0 (range)	17-22 (range)	0.91-0.93 (range)
Somatoform disorders	1[55]	0.08 (0.05-0.11)	1.0 (1.0-1.0)	12 (7.8-18)	0.99 (0.99-1.0)
Psychotic disorders	1[55]	0.19 (0.15-0.25)	1.0 (1.0-1.0)	11 (8.5-14)	0.98 (0.98-0.99)
Any mood disorder	1[55]	0.55 (0.53-0.56)	0.91 (0.91-0.91)	6.0 (5.8-6.2)	0.50 (0.45-0.52)
Any anxiety disorder	1[55]	0.29 (0.27-0.31)	0.95 (0.95-0.95)	5.3 (5-5.6)	0.75 (0.74-0.77)
Past substance-use disorder, other than opioid[a]	2[55,56]	0.14-0.58 (range)	0.95-0.98 (range)	4.2-17 (range)	0.44-0.88 (range)
2+ mental health disorders[a]	1[55]	...	...	2.8-5.3	...
1 mental health disorder[a]	1[55]	...	...	1.3-1.9	...
"0" mental health disorder[a]	1[55]	...	...	0.65-0.72	...
**Prescription characteristics**					
Concomitant medication:	1[55]				
Atypical antipsychotic	1[55]	0.24 (0.22-0.25)	0.10 (0.10-0.10)	17 (15-18)	0.77 (0.76-0.79)

Finding	Studies, Reference #	Sensitivity (95% CI)	Specificity (95% CI)	LR positive (95% CI)	LR negative (95% CI)
Anxiolytic (Buspirone Hydrochloride)	1[55]	0.08 (0.07-0.09)	0.99 (0.99-0.99)	7.3 (6.5-8.3)	0.93 (0.92-0.94)
Tricyclics	1[55]	0.40 (0.38-0.06)	0.92 (0.92-0.92)	5.1 (4.8-5.3)	0.66 (0.64-0.68)
Anticonvulsant	1[55]	0.34 (0.32-0.35)	0.93 (0.93-0.93)	5.0 (4.8-5.3)	0.71 (0.69-0.73)
Other antidepressants	1[55]	0.45 (0.44-0.47)	0.88 (0.88-0.88)	3.8 (3.7-4.0)	0.62 (0.60-0.64)
Benzodiazepine	1[55]	0.53 (0.51-0.54)	0.81 (0.81-0.81)	2.7 (2.6-2.8)	0.59 (0.58-0.61)
Any opioid, i.e., all schedule types[a,#]	1[56]	0.05-0.06 (range)	0.99-0.99 (range)	3.5-4.9 (range)	0.95-0.96 (range)
Opioid dose >120mg/day[a]	1[56]	0.20-0.21 (range)	0.94-0.94 (range)	3.2-3.4 (range)	0.85-0.85 (range)
Opioid type: Schedule II long and short-acting[a]	1[56]	0.07-0.08 (range)	0.97-0.98 (range)	2.8-3.2 (range)	0.95-0.95 (range)
Opioid type: Schedule II long-acting[a]	1[56]	0.14-0.14 (range)	0.95-0.95 (range)	2.8-2.9 (range)	0.90-0.91 (range)

[#]Patients received at least 30 days supply of any opioid, i.e., Schedule III or IV AND short-acting schedule II AND long-acting schedule II opioids within a 6-month period. [a] The LR range is derived from two separate databases described in this study.[56] Any mental health disorder was derived from the presence of adjustment disorder, anxiety disorder, mood disorder, personality disorder, and miscellaneous disorders (such as an eating disorder or somatoform disorder). For results on an ordinal scale (0, 1, 2 mental health disorders) the sensitivity, specificity, and LR- no longer apply. The LR represents the LR at increasing numbers of mental health disorders from 0 to ≥2.

eTable 8. Clinical Criterion Standards for opioid use disorder in pain management among the studies included in the review

Standard	Definition
**DSM III**	**Dependence:**<br>1. *Either tolerance or withdrawal*<br>*(For alcohol and tobacco dependence, either pathological use or impairment in social or occupational functioning is also required)*<br><br>**Abuse:**<br>1. *Pattern of pathological use*<br>2. *Impairment in social or occupational functioning due to substance use*<br>3. *Minimal duration of disturbance of at least one month*
**DSM III-R**	**Dependence:**<br>A. *3 out of 9 symptoms*; symptoms have equal weight*<br>B. *Duration of some symptoms for at least 1 month of symptoms occurred repeatedly over a longer period of time*<br>**(1) Taking substance in larger amounts or over longer period than intended.*<br>*(2) Persistent desire or unsuccessful efforts to cut down or control use.*<br>*(3) Spending a great deal of time to get or use the substance, or recover from its after effects.*<br>*(4) Frequent intoxication or withdrawal when expected to fulfill major obligations.*<br>*(5) Giving up activities for substance use.*<br>*(6) Continuing to use despite problems.*<br>*(7) Tolerance.*<br>*(8) Withdrawal.*<br>*(9) Using substance to relieve or avoid withdrawal symptoms.*<br><br>**Abuse:** *One of the following:*<br>1. *Continued use despite knowledge of having a persistent or recurrent social, occupational, psychological, or physical problem that is caused or exacerbated by use of the psychoactive substance*<br>2. *Recurrent use in situations in which use is physically hazardous*
**DSM IV**	**Dependence:** *Three or more of the following, occurring at any time in the same 12-month period:*<br>*1.Tolerance, as defined by either of the following:*<br>*a. A need for markedly increased amounts of the substance to achieve intoxication or desired effect.*<br>*b. Markedly diminished effect with continued use of the same amount of the substance.*<br>*2.Withdrawal, as manifested by either of the following:*<br>*a. The characteristic withdrawal syndrome for the substance (refer to criteria A and B of the criteria sets for*<br>*Withdrawal from the specific substances).*<br>*b. The same (or a closely related) substance is taken to relieve or avoid withdrawal symptoms.*<br>*3.The substance is often taken in larger amounts or over a longer period than was intended.*<br>*4.There is a persistent desire or unsuccessful efforts to cut down or control substance use.*<br>*5.A great deal of time is spent in activities necessary to obtain the substance, use the substance, or recover from its effects.*<br>*6. Important social, occupational, or recreational activities are given up or reduced because of substance use.*<br>*7.The substance use is continued despite knowledge of having a persistent or recurrent physical or psychological problem that is likely to have been caused or exacerbated by the substance*<br><br>**Abuse:** *One or more of the following, occurring within a 12-month period:*<br>*1.Recurrent substance use resulting in a failure to fulfill major role obligations at work, school, or home.*<br>*2.Recurrent substance use in situations in which it is physically hazardous.*

	3.Recurrent substance-related legal problems<br>*4. Continued substance use despite having persistent or recurrent social or interpersonal problems caused or exacerbated by the effects of the substance.*
**ICD 10**	**Dependence:** *Three of the following:*<br>*1. A strong desire or sense of compulsion to use a substance or substances*<br>*2. Evidence of impaired capacity to control the use of a substance or substances. This may relate to difficulties in avoiding initial use, difficulties in terminating use, or problems about controlling levels of use*<br>*3. A withdrawal state or use of the substance to relieve or avoid withdrawal symptoms, and subjective awareness of the effectiveness of such behavior*<br>*4. Evidence of tolerance to the effects of the substance*<br>*5. Progressive neglect of alternative pleasures, behaviors, or interests in favor of substance use*<br>*6. Persisting with substance use despite clear evidence of harmful consequences*<br><br>**Harmful use:**<br>*1. Clear evidence that the use of a substance or substances was responsible for causing actual psychological or physical harm to the user*
**UDS**	*Urine drug screen conducted in a specialized centre or a hospital. Common methods to detect particular drugs or metabolites include immunoassay and gas-chromatography mass spectrometry.*
DSM = Diagnostic and Statistical Manual; ICD = International Classification of Diseases; UDS = Urine Drug Screen.	

eReferences

1. Haynes RB, McKibbon KA, Wilczynski NL, Walter SD, Werre SR. Optimal search strategies for retrieving scientifically strong studies of treatment from Medline: analytical survey. *BMJ.* 2005;330(7501):1179.
2. Hojsted J, Nielsen PR, Guldstrand SK, Frich L, Sjogren P. Classification and identification of opioid addiction in chronic pain patients. *Eur J Pain.* 2010;14(10):1014-1020.
3. Strain E, Saxon A, Hermann R. Opioid use disorder: Epidemiology, pharmacology, clinical manifestations, course, screening, assessment, and diagnosis. In: Uptodate Literature review current through: Oct; 2015.
4. Mathers C, Vos E, Stevenson C, Begg S. The Australian burden of disease study: Measuring the loss of health from diseases, injuries and risk factors. *Medical Journal of Australia.* 2000;172(12):592 - 596.
5. McGrath TA, Alabousi M, Skidmore B, et al. Recommendations for reporting of systematic reviews and meta-analyses of diagnostic test accuracy: a systematic review. *Systematic Reviews.* 2017;6:194.
6. Sharifabadi AD, McInnes MDF, Bossuyt PMM. PRISMA-DTA: An Extension of PRISMA for Reporting of Diagnostic Test Accuracy Systematic Reviews. *Clinical Chemistry.* 2018;64(6):985-986.
7. Whiting P, Rutjes AW, Reitsma JB, Bossuyt PM, Kleijnen J. The development of QUADAS: a tool for the quality assessment of studies of diagnostic accuracy included in systematic reviews. *BMC medical research methodology.* 2003;3:25.
8. Whiting PF, Weswood ME, Rutjes AW, Reitsma JB, Bossuyt PN, Kleijnen J. Evaluation of QUADAS, a tool for the quality assessment of diagnostic accuracy studies. *BMC medical research methodology.* 2006;6:9.
9. Minozzi S, Amato L, Davoli M. Development of dependence following treatment with opioid analgesics for pain relief: a systematic review. *Addiction.* 2013;108(4):688-698.
10. Voon P, Karamouzian M, Kerr T. Chronic pain and opioid misuse: a review of reviews. *Substance Abuse Treatment, Prevention, and Policy.* 2017;12(1):36.
11. Sehgal N, Manchikanti L, Smith HS. Prescription opioid abuse in chronic pain: a review of opioid abuse predictors and strategies to curb opioid abuse. *Pain physician.* 2012;15(3 Suppl):ES67-92.
12. Borenstein M, Hedges L, Higgins J, Rothstein H. Comprehensive Meta-Analysis Version 3. In. Englewood, NJ: Biostat; 2013.
13. Wu SM, Compton P, Bolus R, et al. The addiction behaviors checklist: validation of a new clinician-based measure of inappropriate opioid use in chronic pain. *J Pain Symptom Manage.* 2006;32(4):342-351.
14. Chabal C, Erjavec MK, Jacobson L, Mariano A, Chaney E. Prescription opiate abuse in chronic pain patients: clinical criteria, incidence, and predictors. *Clin J Pain.* 1997;13(2):150-155.
15. Meltzer EC, Rybin D, Saitz R, et al. Identifying prescription opioid use disorder in primary care: diagnostic characteristics of the Current Opioid Misuse Measure (COMM). *Pain.* 2011;152(2):397-402.
16. Butler SF, Budman SH, Fernandez KC, et al. Development and validation of the Current Opioid Misuse Measure. *Pain.* 2007;130(1-2):144-156.
17. Butler SF, Budman SH, Fanciullo GJ, Jamison RN. Cross validation of the current opioid misuse measure to monitor chronic pain patients on opioid therapy. *Clin J Pain.* 2010;26(9):770-776.
18. Witkin LR, Diskina D, Fernandes S, Farrar JT, Ashburn MA. Usefulness of the opioid risk tool to predict aberrant drug-related behavior in patients receiving opioids for the treatment of chronic pain. *Journal of opioid management.* 2013;9(3):177-187.
19. Webster LR, Webster RM. Predicting aberrant behaviors in opioid-treated patients: preliminary validation of the Opioid Risk Tool. *Pain Med.* 2005;6(6):432-442.

20. Jones T, Lookatch S, Moore T. Validation of a new risk assessment tool: the Brief Risk Questionnaire. *J Opioid Manag.* 2015;11(2):171-183.
21. Passik SD, Kirsh KL, Whitcomb L, et al. A new tool to assess and document pain outcomes in chronic pain patients receiving opioid therapy. *Clin Ther.* 2004;26(4):552-561.
22. Dowling LS, Gatchel RJ, Adams LL, Stowell AW, Bernstein D. An evaluation of the predictive validity of the Pain Medication Questionnaire with a heterogeneous group of patients with chronic pain. *J Opioid Manag.* 2007;3(5):257-266.
23. Hojsted J, Nielsen PR, Kendall S, Frich L, Sjogren P. Validation and usefulness of the Danish version of the Pain Medication Questionnaire in opioid-treated chronic pain patients. *Acta Anaesthesiol Scand.* 2011;55(10):1231-1238.
24. Buelow AK, Haggard R, Gatchel RJ. Additional validation of the pain medication questionnaire in a heterogeneous sample of chronic pain patients. *Pain Pract.* 2009;9(6):428-434.
25. Holmes CP, Gatchel RJ, Adams LL, et al. An opioid screening instrument: long-term evaluation of the utility of the Pain Medication Questionnaire. *Pain Pract.* 2006;6(2):74-88.
26. Adams LL, Gatchel RJ, Robinson RC, et al. Development of a self-report screening instrument for assessing potential opioid medication misuse in chronic pain patients. *J Pain Symptom Manage.* 2004;27(5):440-459.
27. Banta-Green CJ, Von Korff M, Sullivan MD, Merrill JO, Doyle SR, Saunders K. The prescribed opioids difficulties scale: a patient-centered assessment of problems and concerns. *Clin J Pain.* 2010;26(6):489-497.
28. Compton P, Darakjian J, Miotto K. Screening for addiction in patients with chronic pain and "problematic" substance use: evaluation of a pilot assessment tool. *J Pain Symptom Manage.* 1998;16(6):355-363.
29. Compton PA, Wu SM, Schieffer B, Pham Q, Naliboff BD. Introduction of a self-report version of the Prescription Drug Use Questionnaire and relationship to medication agreement noncompliance. *J Pain Symptom Manage.* 2008;36(4):383-395.
30. Wasan AD, Butler SF, Budman SH, Benoit C, Fernandez K, Jamison RN. Psychiatric history and psychologic adjustment as risk factors for aberrant drug-related behavior among patients with chronic pain. *Clin J Pain.* 2007;23(4):307-315.
31. Knisely JS, Wunsch MJ, Cropsey KL, Campbell ED. Prescription Opioid Misuse Index: a brief questionnaire to assess misuse. *J Subst Abuse Treat.* 2008;35(4):380-386.
32. Butler SF, Budman SH, Fernandez K, Jamison RN. Validation of a screener and opioid assessment measure for patients with chronic pain. *Pain.* 2004;112(1-2):65-75.
33. Akbik H, Butler SF, Budman SH, Fernandez K, Katz NP, Jamison RN. Validation and clinical application of the Screener and Opioid Assessment for Patients with Pain (SOAPP). *J Pain Symptom Manage.* 2006;32(3):287-293.
34. Brown J, Setnik B, Lee K, et al. Assessment, stratification, and monitoring of the risk for prescription opioid misuse and abuse in the primary care setting. *J Opioid Manag.* 2011;7(6):467-483.
35. Butler SF, Budman SH, Fernandez KC, Fanciullo GJ, Jamison RN. Cross-Validation of a Screener to Predict Opioid Misuse in Chronic Pain Patients (SOAPP-R). *J Addict Med.* 2009;3(2):66-73.
36. Butler SF, Fernandez K, Benoit C, Budman SH, Jamison RN. Validation of the revised Screener and Opioid Assessment for Patients with Pain (SOAPP-R). *J Pain.* 2008;9(4):360-372.
37. Belgrade MJ, Schamber CD, Lindgren BR. The DIRE score: predicting outcomes of opioid prescribing for chronic pain. *J Pain.* 2006;7(9):671-681.
38. Coambs RB, Jarry JL, Santhiapillai AC, Abrahamsohn RV, Atance CM. The SISAP: A New Screening Instrument for Identifying Potential Opioid Abusers in the Management of Chronic Nonmalignant Pain Within General Medical Practice. Pain Research and Management. 1996;1(3):155-162.
39. Atluri SL, Sudarshan G. Development of a screening tool to detect the risk of inappropriate prescription opioid use in patients with chronic pain. *Pain Physician.* 2004;7(3):333-338.

40. Friedman R, Li V, Mehrotra D. Treating pain patients at risk: evaluation of a screening tool in opioid-treated pain patients with and without addiction. *Pain Med.* 2003;4(2):182-185.
41. Brenton A, Richeimer S, Sharma M, et al. Observational study to calculate addictive risk to opioids: a validation study of a predictive algorithm to evaluate opioid use disorder. *Pharmgenomics Pers Med.* 2017;10:187-195.
42. Leonardi C, Vellucci R, Mammucari M, Fanelli G. Opioid risk addiction in the management of chronic pain in primary care: the addiction risk questionnaire. *Eur Rev Med Pharmacol Sci.* 2015;19(24):4898-4905.
43. Larance B, Bruno R, Lintzeris N, et al. Development of a brief tool for monitoring aberrant behaviours among patients receiving long-term opioid therapy: The Opioid-Related Behaviours In Treatment (ORBIT) scale. *Drug Alcohol Depend.* 2016;159:42-52.
44. Jones T, Moore T. Preliminary data on a new opioid risk assessment measure: the Brief Risk Interview. *J Opioid Manag.* 2013;9(1):19-27.
45. Jones T, Lookatch S, Grant P, McIntyre J, Moore T. Further validation of an opioid risk assessment tool: the Brief Risk Interview. *J Opioid Manag.* 2014;10(5):353-364.
46. Averill LA, Averill CL, Staley LA, Ozawa-Kirk JL, Kauwe JS, Henrie-Barrus P. The Opioid Abuse Risk Screener predicts aberrant same-day urine drug tests and 1-year controlled substance database checks: A brief report. *Health Psychol Open.* 2017;4(2):2055102917748459.
47. Fleming MF, Davis J, Passik SD. Reported lifetime aberrant drug-taking behaviors are predictive of current substance use and mental health problems in primary care patients. *Pain Med.* 2008;9(8):1098-1106.
48. Manchikanti L, Singh V, Damron KS, Beyer CD, Pampati V. Screening for controlled substance abuse in interventional pain management settings: evaluation of an assessment tool. *Pain Physician.* 2003;6(4):425-433.
49. Manchikanti L, Pampati V, Damron KS, McManus CD. Evaluation of variables in illicit drug use: does a controlled substance abuse screening tool identify illicit drug use? *Pain Physician.* 2004;7(1):71-75.
50. Jamison RN, Martel MO, Huang CC, Jurcik D, Edwards RR. Efficacy of the Opioid Compliance Checklist to Monitor Chronic Pain Patients Receiving Opioid Therapy in Primary Care. *J Pain.* 2016;17(4):414-423.
51. Jamison RN, Martel MO, Edwards RR, Qian J, Sheehan KA, Ross EL. Validation of a brief Opioid Compliance Checklist for patients with chronic pain. *J Pain.* 2014;15(11):1092-1101.
52. Michna E, Ross EL, Hynes WL, et al. Predicting aberrant drug behavior in patients treated for chronic pain: importance of abuse history. *J Pain Symptom Manage.* 2004;28(3):250-258.
53. Pouget ER, Bennett AS, Elliott L, et al. Development of an opioid-related Overdose Risk Behavior Scale (ORBS). *Subst Abus.* 2017;38(3):239-244.
54. Carra G, Crocamo C, Humphris G, et al. Engagement in the Overdose RIsk InfOrmatioN (ORION) e-Health Tool for Opioid Overdose Prevention and Self-Efficacy: A Preliminary Study. *Cyberpsychol Behav Soc Netw.* 2017;20(12):762-768.
55. Cochran BN, Flentje A, Heck NC, et al. Factors predicting development of opioid use disorders among individuals who receive an initial opioid prescription: mathematical modeling using a database of commercially-insured individuals. *Drug Alcohol Depend.* 2014;138:202-208.
56. Edlund MJ, Martin BC, Fan MY, Devries A, Braden JB, Sullivan MD. Risks for opioid abuse and dependence among recipients of chronic opioid therapy: results from the TROUP study. *Drug Alcohol Depend.* 2010;112(1-2):90-98.
57. Clarke H, Soneji N, Ko DT, Yun L, Wijeysundera DN. Rates and risk factors for prolonged opioid use after major surgery: population based cohort study. *BMJ.* 2014;348:g1251.
58. Hooten WM, St Sauver JL, McGree ME, Jacobson DJ, Warner DO. Incidence and Risk Factors for Progression From Short-term to Episodic or Long-term Opioid Prescribing: A Population-Based Study. *Mayo Clin Proc.* 2015;90(7):850-856.
